# Small molecule inhibition of the mitochondrial lipid transfer protein STARD7 attenuates influenza viral replication

**DOI:** 10.1371/journal.ppat.1013914

**Published:** 2026-09-11

**Authors:** Shipra Sharma, Oyahida Khatun, Jihaan Adonis, Shaochen You, Kevin Hartenbower, Vedita Anand Singh, Namir Shaabani, Saikat De, Emi R. Matsuo, Rachel Y. Sattler, Jonathan A. Covel, Kris M. White, Laura Martin-Sancho, Malina A. Bakowski, Dylan J. Mendonsa, Ayush Mehta, Noura S. Yassir, Steven H. Olson, Naoko Matsunaga, Adolfo García-Sastre, Michael J. Bollong, Nisar A. Farhat, Megan L. Shaw, Sumit K. Chanda

**Affiliations:** 1 Department of Immunology and Microbiology, The Scripps Research Institute, La Jolla, California, United States of America; 2 Calibr, a Division of The Scripps Research Institute, La Jolla, California, United States of America; 3 Department of Medical Biosciences, University of the Western Cape, Bellville, Cape Town, South Africa; 4 Department of Chemistry, Scripps Research, San Diego, California, United States of America; 5 Conrad Prebys Center for Chemical Genomics, Sanford Burnham Prebys Medical Discovery Institute, La Jolla, California, United States of America; 6 Department of Microbiology, Icahn School of Medicine at Mount Sinai, New York, New York, United States of America; 7 Department of Medicine, Division of Infectious Diseases, Icahn School of Medicine at Mount Sinai, New York, New York, United States of America; 8 Global Health and Emerging Pathogens Institute, Icahn School of Medicine at Mount Sinai, New York, New York, United States of America; 9 The Tisch Cancer Institute, Icahn School of Medicine at Mount Sinai, New York, New York, United States of America; 10 Department of Pathology, Molecular and Cell-Based Medicine, Icahn School of Medicine at Mount Sinai, New York, New York, United States of America; 11 The Icahn Genomics Institute, Icahn School of Medicine at Mount Sinai, New York, New York, United States of America; 12 Division of Medical Virology, Faculty of Medicine and Health Sciences, Stellenbosch University, Cape Town, South Africa; Dalhousie University, CANADA

## Abstract

The increasing appearance of drug-resistant and zoonotic influenza strains highlights an urgent need for host-directed antivirals that offer broad-spectrum activity and a higher barrier to resistance. Here, we describe the characterization of M4, a small-molecule identified from a high-throughput screen that potently inhibits influenza A and B viruses. Mechanistic studies reveal that M4 suppresses influenza virus replication by preventing formation of export-competent viral ribonucleoprotein (vRNP) complexes in the nucleus. Chemoproteomic profiling identified the lipid transfer protein STARD7 as the primary cellular target, and genetic depletion of STARD7 phenocopies the antiviral effects of M4. Additional studies localized the M4 binding site to cysteine 302 within the lipid-binding domain of STARD7, supporting a model in which STARD7-dependent lipid transfer activity promotes efficient vRNP assembly and nuclear export. Combining M4 with baloxavir enhances antiviral efficacy in a murine infection model, providing in vivo support for a host-directed therapeutic strategy. Together, these results identify STARD7 as a metabolic checkpoint licensing vRNP nuclear export and they establish a proof of concept for therapeutic intervention with small molecule inhibitors.

## Author summary

Influenza viruses continue to cause widespread illness and pose a pandemic threat, in part because existing antiviral drugs can lose effectiveness as the virus evolves resistance. To address this challenge, we focused on therapies that target host cell processes required for viral replication, rather than viral proteins. In this study, we describe a small molecule, M4, that inhibits replication of both influenza A and B viruses by blocking a critical step in the viral life cycle. We found that M4 acts on a host protein called STARD7, which aids in the intracellular movement of lipids. When STARD7 is inhibited, the components of the influenza viral ribonucleoprotein (vRNP) complex become trapped in the nucleus, preventing the virus from completing its replication cycle. Disrupting STARD7 genetically produces the same effect, confirming that this host protein is important for influenza replication. These findings point to a previously unrecognized gating mechanism that controls assembly and nuclear export of the vRNP complex during infection.Although M4 alone showed limited activity in animals, combining it with an existing antiviral drug improved antiviral efficacy. Together, our results reveal a host pathway that influenza viruses rely on, and they support host-directed combination approaches to strengthen antiviral treatment and help counter drug resistance.

## Introduction

Influenza A viruses (IAV) are recognized for causing seasonal epidemics and occasional pandemics [1]. These events result in significant morbidity and mortality, particularly among vulnerable populations, often straining healthcare resources [2]. Influenza A virus subtypes H1, H2, and H3 have caused four historic pandemics, and recent outbreaks involving the H5, H7, and H9 IAV subtypes indicate their potential for zoonotic transmission and associated pandemic risk [3]. Of particular concern is avian H5N1 IAV that is now endemic among wild birds globally, resulting in multiple outbreaks in poultry and most recently also in dairy cows [4]. Although human infections have been rare thus far, and no sustained human-to-human transmission has been reported, the high number of H5N1 viruses circulating in nature and evidence of adaptation and limited transmission in several mammals (marine mammals, minks, and cows) raise increased concerns on their human pandemic potential [5,6]

Regardless of strain, the standard of care treatments for influenza infection are direct-acting antivirals. The neuraminidase (NA) inhibitors- oseltamivir (Tamiflu), zanamivir and peramivir- are approved for the treatment of influenza A and B and act by preventing efficient release of virions from infected cells (McKimm‐ [7]. Baloxavir marboxil (BXA) targets the polymerase acidic (PA) endonuclease subunit of the influenza virus polymerase complex, thereby disrupting viral RNA transcription and replication and was approved by US FDA for the treatment of acute uncomplicated influenza [8], [9]). Favipiravir is a nucleoside analog inhibitor of viral RNA-dependent RNA polymerases, suppressing replication of viral genomes, but is currently only approved in Japan for the treatment of pandemic influenza, not seasonal influenza [10]. All of these drugs have been clinically demonstrated to reduce the duration of influenza symptoms and may also limit the spread of the virus to others [11–15]. However, their efficacy can be compromised by the rapid emergence of resistant viral variants. In fact, M2 inhibitors (i.e., amantadine, rimantidine) are no longer clinically useful as all human circulating influenza A viruses are resistant [16]. The emergence and rapid spread of oseltamivir resistant H1N1 influenza A viruses were cause for concern in 2008, but these viruses were replaced by the 2009 H1N1 pandemic virus that is oseltamivir sensitive [17]. Yet, sporadic cases of oseltamivir resistance continue to be identified, including the NA-S247N mutation in 3 human cases of influenza H5N1, which has been reported to reduce susceptibility to oseltamivir [18]. Taken together, these observations show that oseltamivir-resistant viruses can emerge sporadically and persist in circulation. Lastly, baloxavir marboxil is also sensitive to the emergence of resistance mutations, as observed during clinical trials [19]. To address therapeutic challenges for both seasonal influenza and emerging pandemic influenza strains, there is an urgent need for the development of novel antiviral agents, particularly with broad-spectrum activity and a higher barrier to resistance.

Influenza virus replication relies on a significant compendium of host (cellular) proteins to complete an infectious replication cycle, many of which have been comprehensively elucidated though several OMICs-level studies [20–24]. Host-directed therapies against viruses represent a growing area of interest in antiviral research and treatment strategies. Targeting host proteins can reduce the likelihood of the virus developing resistance but may also increase the risk of side effects due to disruption of normal cellular functions [25]. To date, few such therapies have been developed. Most host-directed drugs used in the clinic have immunomodulatory properties that boost natural antiviral responses (e.g., interferon), or that dampen immunopathogenesis due to viral infection (e.g., corticosteroids) [25–27].

We have previously reported a high-throughput screen assessing the activities of approximately 1 million compounds in a cell-based assay of influenza virus replication [28]. In the present study, we characterize one of the active antiviral compounds, M4, which possesses broad-spectrum antiviral activity against several influenza virus subtypes both ex vivo, and in combination with DAAs, in vivo. This, combined with the inability to select for resistance, suggested a host-directed mechanism of antiviral activity. Employing a chemoproteomics approach, we determined that M4 targets STARD7 (StAR-related lipid transfer domain containing 7). Here, we identify STARD7 as a host factor required for efficient influenza virus replication and show that pharmacological inhibition of STARD7 suppresses influenza virus infection by impairing vRNP assembly and nuclear export.

Although the XPO1/CRM1 pathway is known to mediate nuclear export of this complex, it remains unclear how host lipid metabolism, intracellular trafficking machinery, and signaling pathways converge to license formation of the vRNP complex [29]. vRNP nuclear export is essential for productive infection and requires assembly of nuclear vRNPs with the viral proteins M1 and NEP to form an export-competent complex [30]. Notably, influenza hemagglutinin signaling has been shown to activate host kinase cascades, including the Raf–MEK–ERK–RSK axis, which promotes phosphorylation of NP and facilitates its interaction with M1, thereby licensing nuclear export of vRNPs [31]. How these signaling events are governed by cellular processes, including cellular metabolic state, membrane composition, or lipid-transfer pathways, to regulate the IAV nuclear export checkpoint, remains poorly defined. A more mechanistic understanding of how viral cues and host metabolic and signaling pathways intersect to control vRNP assembly and trafficking may reveal new opportunities for therapeutic intervention to block influenza virus replication.

Our findings implicate STARD7, a regulator of phospholipid trafficking, as a critical host factor required for efficient vRNP assembly and nuclear export. M4 engages STARD7 within its lipid-binding pocket, a region essential for phosphatidylcholine transfer and mitochondrial lipid homeostasis, linking disruption of this activity to impaired influenza replication. Together, these findings position STARD7 as a metabolic gatekeeper of vRNP nuclear egress and highlight lipid-handling pathways as tractable targets for host-directed antiviral development.

## Results

### Antiviral activity of M4 against influenza viruses

**Fig 1:**
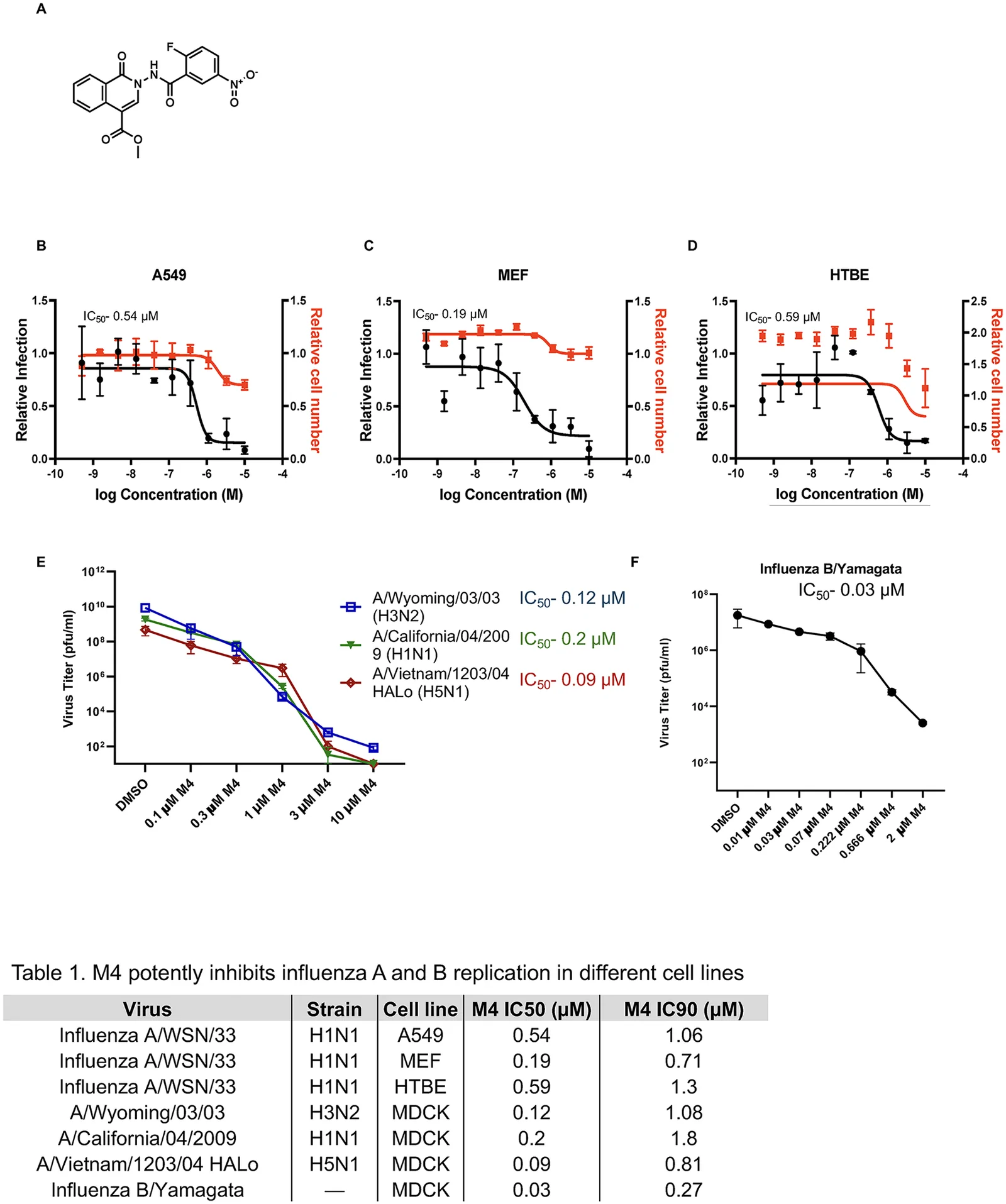
The M4 compound is a broadly-acting influenza virus inhibitor: (A) Chemical structure of M4. **(B)** After pretreatment with M4 for 16 h at the indicated concentrations, A549 cells were infected with influenza A/WSN/33 virus at MOI of 0.1, **(C)** MEF cells were infected at MOI of 0.5 and **(D)** HTBE cells were infected at MOI of 0.25. 48 h post infection, cells were fixed, stained for NP, and analyzed with a high content immunofluorescence imager. Percent infection was calculated as the ratio of anti-NP-stained cells to DAPI stained cells. Data are normalized by the mean for DMSO-treated wells and are shown as means ± SD from three independent experiments. Dose-response curves for infectivity (black) and cell number (red) are shown. **(E-F)** MDCK cells were pre-treated for 16 h with different concentrations of M4 followed by infection with the indicated influenza A subtypes at MOI of 0.1 (E) or influenza B/Yamagata at MOI of 0.2 **(F)**. Supernatants were analyzed at 24h post infection by plaque assay and data are represented as means ±  SD. of three independent experiments. The IC_50_ values are indicated in Table 1.

We have previously reported a high-throughput screen of approximately 1 million small molecules using a recombinant influenza A/WSN/33 virus expressing Renilla luciferase, enabling the capture of inhibitors across all stages of the viral life cycle [28]. One of the most potent compounds identified through this analysis was a small molecule designated as M4 (structure shown in Fig 1A), which exhibited a half-maximal inhibitory concentration (IC50) <0.6 μM, and a selectivity index (SI) >10 against influenza A/WSN/33 (H1N1) virus (WSN) in the human lung epithelial cell line (A549), mouse embryonic fibroblast cells (MEF), and primary human tracheal bronchial epithelial (HTBE) (Fig 1B–D; Table 1). The antiviral activity of M4 against different influenza A virus subtypes and influenza B virus was further confirmed using a multicycle growth assay and plaque assay readout (Fig 1E and 1F, respectively, Table 1). M4 potently inhibited influenza B/Yamagata/16/88 virus with an IC_50_ of 0.03 μM, and an attenuated highly pathogenic avian influenza H5N1 strain (A/Vietnam/1203/04 HALo), with an IC_50_ of 0.09 μM. It was also potent against influenza A/Wyoming/03/03 (H3N2) and influenza A/California/04/2009 (H1N1) viruses, yielding IC_50_s of 0.12 μM and 0.2 μM, respectively. Overall, treatment with 1 μM M4 resulted in a > 1.5-log10 reduction in infectious viral particle release by cells infected for 24 h at MOI of 0.1 with influenza A or influenza B virus (Fig 1E–F).

**Table 1:** M4 potently inhibits influenza A and B replication in different cell lines.

Virus	Strain	Cell line	M4 IC50 (µM)	M4 IC90 (µM)
Influenza A/WSN/33	H1N1	A549	0.54	1.06
Influenza A/WSN/33	H1N1	MEF	0.19	0.71
Influenza A/WSN/33	H1N1	HTBE	0.59	1.3
A/Wyoming/03/03	H3N2	MDCK	0.12	1.08
A/California/04/2009	H1N1	MDCK	0.2	1.8
A/Vietnam/1203/04	H5N1	MDCK	0.09	0.81
Influenza B/Yamagata		MDCK	0.03	0.27

### M4 inhibits nuclear export of the vRNP complex

**Fig 2:**
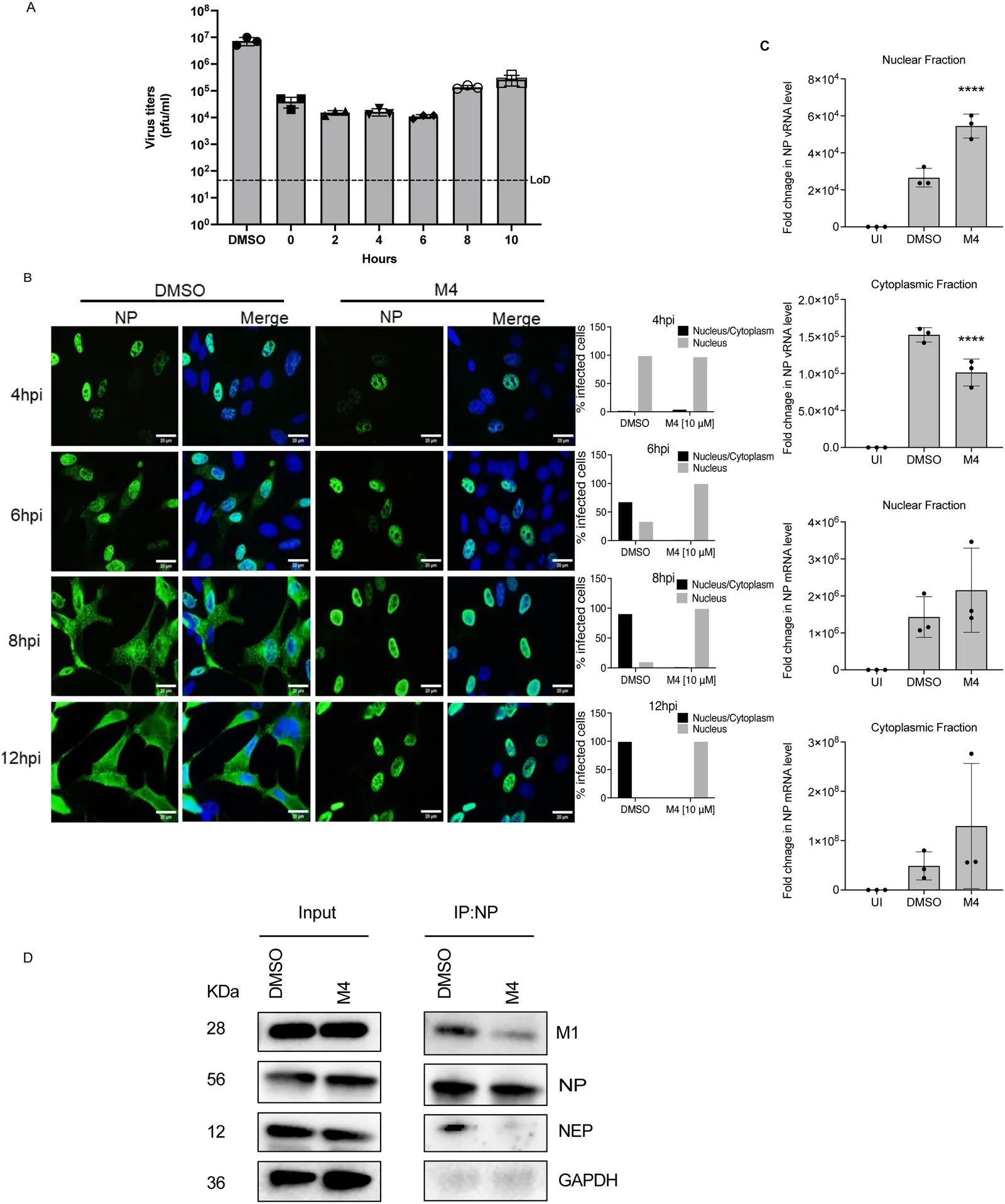
M4 inhibits post entry steps and causes nuclear accumulation of the viral RNP complex: (A) Time-of-addition assay. A549 cells were treated with 3 µM M4 at the indicated times post infection with influenza A/WSN/33 virus at MOI of 0.1. Titers were quantified by plaque assay at 24 h post infection. Statistical significance was determined with a one-way ANOVA. ****p < 0.0001, ***p < 0.001. **(B)** Subcellular localization of NP in M4 treated cells. MDCK cells were treated with DMSO or M4 (10 µM) and infected with influenza A/WSN/33 (MOI 5). Cells were immunostained with anti-NP antibody at 4, 6, 8, and 12 hpi, and nuclei visualised with DAPI. Scale bar = *20 µm*. For quantification, 5 fields of view were randomly selected for each condition, and the localization of NP was recorded as present in the “nucleus” or “nucleus/cytoplasm”. **(C)** Subcellular localization of vRNA and mRNA. A549 cells were pretreated for 16 h with DMSO or 1 µM M4 followed by mock infection (UI) or infection with influenza virus A/WSN/33 at an MOI of 1 for 12 h. The cells were then subjected to cytoplasmic-nuclear fractionation, followed by RNA isolation from each fraction. The relative nuclear-cytoplasmic distribution of influenza virus NP vRNA (upper panels) NP mRNA (lower panels), was assessed from both subcellular fractions followed by RT-qPCR analysis. Viral RNA abundance in the cytoplasmic fraction was normalized to GAPDH and for the nuclear fraction, with U6 snRNA. The resulting data, relative to that in mock-infected cells, were graphed. Statistical significance was determined with a one-way ANOVA. ****p < 0.0001, ***p < 0.001. **(D)** Immunoprecipitation of vRNP. A549 cells were pre-treated with DMSO or M4 (5 µM) and infected with influenza A/WSN/33 virus (MOI 3). At 8 h post infection, cell lysates were subjected to immunoprecipitation using an anti-NP antibody. Input and NP-immunoprecipitated fractions were analyzed by SDS–PAGE and immunoblotting for viral proteins M1, NEP, and NP. GAPDH serves as a control for nonspecific associations. Representative immunoblots are shown.

To ascertain the stage of the viral life cycle inhibited by M4, we performed a time of addition assay. M4 (at>IC_90_ concentration) inhibited replication of WSN by 2 logs or more when added between 0 and 6 h post-infection (hpi) (Fig 2A). This indicates that M4 acts at post-entry stage in the replication cycle. Furthermore, using HA/NA pseudotyped vesicular stomatitis virus particles, we further confirmed that M4 does not inhibit viral entry (S1A Fig).

To investigate a potential post-entry mechanism, we asked whether M4 perturbs intracellular trafficking of viral ribonucleoprotein complexes. We therefore visualized the effect of M4 on trafficking of the viral nucleoprotein (NP). Cells were pretreated with M4 (3 μM), infected with WSN at a high multiplicity, and fixed and stained with an anti-NP antibody at 4, 6, 8, and 12 h post-infection in order to track the movement of NP during a single round of infection (Fig 2B). In DMSO treated cells, NP exhibits predominantly nuclear staining at the early timepoints, but becomes increasingly cytoplasmic by 8–12 h post infection as the vRNP is exported from the nucleus for assembly into virions. By contrast, in M4 treated cells, NP staining remains nuclear, even at the later timepoints. To determine if this effect is specific for NP or extends to other components of the vRNP, immunofluorescence staining of NP, PB1, PB2, and PA was performed at 24 h post infection in the presence of DMSO or M4 (S2A Fig). Over 90% of cells treated with M4 exhibited exclusively nuclear staining of all vRNP proteins, whereas cells treated with DMSO exhibited a diffuse staining, indicating their presence in both the nucleus and the cytoplasm. Similarly, staining for the viral M1 protein over a single infection cycle in the presence of M4, showed that M1, which associates with the vRNP complex to facilitate nuclear export, also remains trapped in the nucleus (S2B Fig). Finally, we examined the localization of influenza virus vRNA and mRNA in M4-treated cells. A549 cells were pre-treated with M4 followed by infection with WSN for 12 h, allowing only one cycle of replication. Subsequently, subcellular fractionation was performed, and RNA was isolated from cytoplasmic and nuclear fractions. The quantification of negative-sense NP vRNA and positive-sense NP mRNA, in both the cytoplasm and nucleus, was evaluated using strand-specific reverse transcription quantitative polymerase chain reaction (RT-qPCR). Cells pre-treated with M4 exhibited a two-fold enrichment of nuclear NP vRNA, compared to DMSO-treated cells (Fig 2C). Simultaneously, the relative abundance of cytoplasmic NP vRNA was significantly reduced. Nuclear and cytoplasmic NP mRNA levels remained unaffected in M4 treated cells (Fig 2C). Immunoblotting for nuclear and cytoplasmic marker proteins confirmed the purity of the subcellular fractions (S2C Fig). Taken together, these findings indicate that M4 causes nuclear retention of the influenza vRNP complex, with both RNA and protein components failing to reach the cytoplasmic compartment. Furthermore, time-course analyses showed only a modest, transient reduction in viral protein and vRNA levels at 8 hpi following M4 treatment that was not statistically significant and resolved by 12–24 hpi, whereas viral mRNA levels remained unchanged throughout the course of infection (S2D and S2E Fig). To assess whether the antiviral activity of M4 resulted from nonspecific effects on host cellular function, we evaluated its activity against dengue virus (DENV) and poliovirus. M4 exhibited no detectable antiviral activity against either virus, further supporting that its antiviral effect is not attributable to a broad inhibition of viral gene expression or protein synthesis (S2F and S2G Fig). Notably, RanBP1, a host cellular protein that is exported out of the nucleus through the CRM1 complex [31], did not display nuclear retention following M4 treatment (S2A Fig). As an additional control, we investigated the cellular distribution of STAT1, which translocates to the nucleus upon interferon (IFN) stimulation, followed by re-export into the cytoplasm [32]. This nuclear export is CRM1-dependent and therefore leptomycin B (LMB)-sensitive. Using a STAT1-GFP fusion, the movement of STAT1 in response to IFN-β was monitored in live cells (S2H Fig). At 1h post IFN-β stimulation in DMSO, M4, and LMB-treated cells, STAT1-GFP was located in the nucleus. By 5 h post-IFNβ stimulation, STAT1-GFP was located both in the nucleus and cytoplasm of DMSO and M4 treated cells, whereas it was primarily localized in the nuclei of LMB-treated cells. These data show that M4 does not act like LMB, suggesting that M4 likely does not target the nuclear export of influenza virus vRNPs by directly inhibiting the CRM1-mediated export pathway. Since it is known that CRM1 is required for vRNP nuclear export [30], our results suggest that M4 specifically targets steps preceding CRM1-dependent export that is unique to vRNP transit.

To investigate whether M4 interferes with the steps that facilitate vRNP export prior to CRM1-mediated export, we examined the association of NP with the viral proteins M1 and NEP, which coordinate formation of export-competent vRNP complexes. In the presence of M4, NP showed markedly reduced association with both proteins (Fig 2D). We confirmed that the immunoprecipitated NP represented bona fide vRNP-associated NP by detecting copurifying vRNA (S2I Fig). These findings support a model in which M4 reduces the assembly of export-ready vRNP complexes by directly or indirectly diminishing NP interactions with M1 and NEP. This would be consistent with the M4-mediated specific inhibition of vRNP nuclear export as compared to other CRM-1 exported proteins.

### M4 is a host-directed antiviral that targets STARD7

We next sought to determine if M4 is a direct-acting or a host-directed antiviral. A distinguishing feature between these two classes of inhibitors is that RNA viruses usually can rapidly acquire resistance to direct-acting antivirals [33]. WSN was serially passaged in A549 cells in the presence of M4 (IC_90_), baloxavir marboxil (BXA, IC_90_) as a direct-acting antiviral control, or DMSO (as a control) (S3A Fig). After 10 passages, the resulting viruses were assessed for antiviral sensitivity by plaque reduction assay. As expected, BXA-passaged virus exhibited reduced susceptibility to inhibition by BXA, whereas M4-passaged virus retained susceptibility to M4 inhibition comparable to that of the DMSO-passaged virus, with no appreciable shift in IC50 (S3B and S3C Fig). This outcome implies that M4 creates a higher barrier to resistance and may act on a host cell component, rather than directly targeting the virus itself.

**Fig 3:**
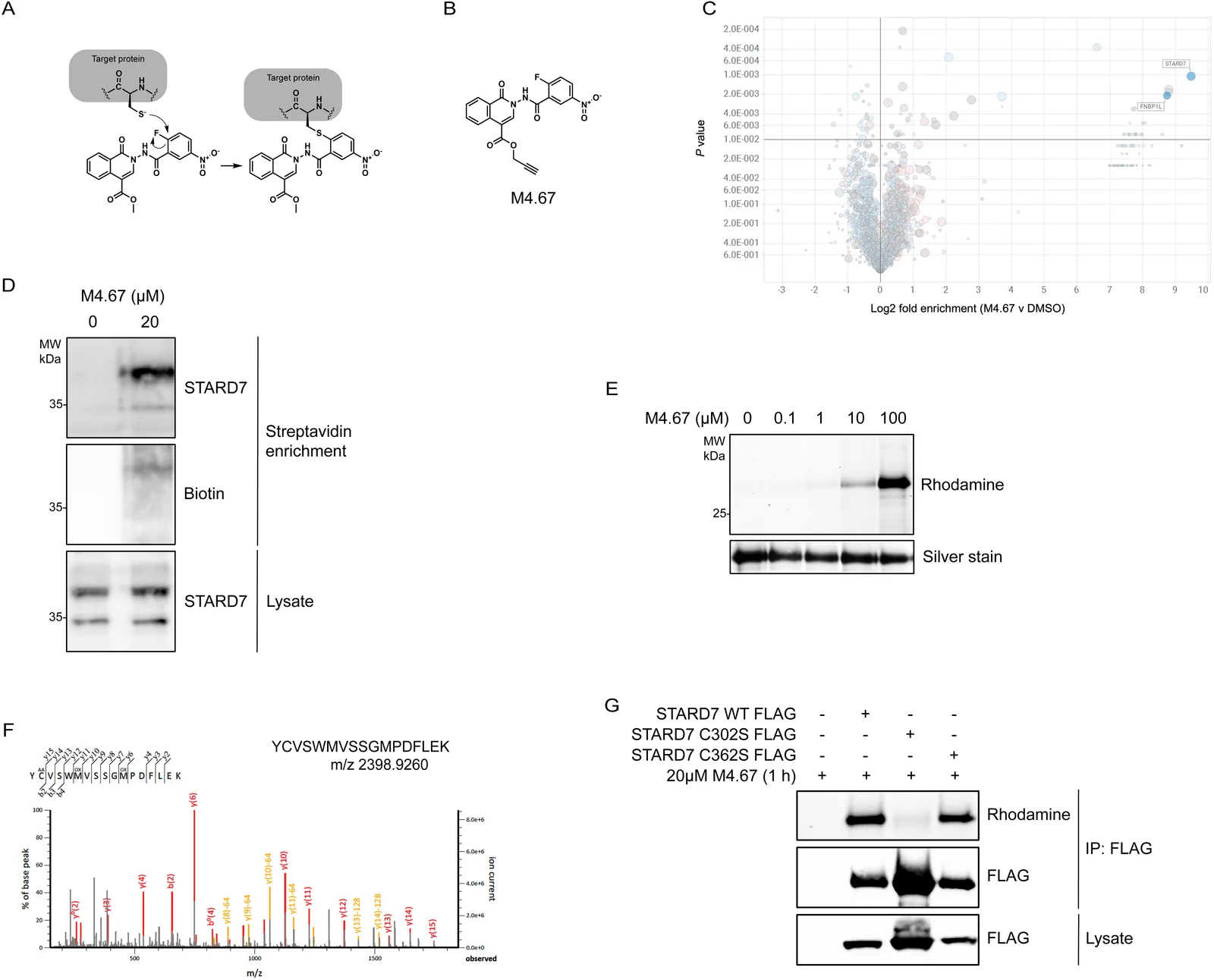
Proteomic enrichment of M4.67 target: (A) Schematic illustrating covalent modification of the target protein by M4. **(B)** Structure of M4.67 probe. **(C)** Scatter plot of fold enrichment and P value of potential cellular targets from HEK293T cells exposed to M4.67 (1 µM) relative to DMSO. **(D)** Representative anti-STARD7 and anti-biotin western blot of M4.67 (20 µM) treated A549 cells after streptavidin enrichment of labeled proteins. **(E)** Representative rhodamine scan and silver stain of M4.67 treated HEK293T cells transfected with STARD7 WT-FLAG transgene after FLAG immunoprecipitation. **(F)** MS/MS spectra of STARD7 peptide containing modified C302 from anti-FLAG immunoprecipitated material from HEK293T cells expressing STARD7 WT FLAG treated with 20 µM M4.67 for 1 **h. (G)** Rhodamine fluorescence scan and anti-FLAG Western blot of anti-FLAG immunoprecipitated content of the indicated FLAG tagged STARD7 transgenes overexpressed in HEK293T cells and exposed to M4.67 (20 μM) for 1 **h.**

We hypothesized that M4 may covalently bind its target protein due to the presence of its 2-fluoro-5-nitrobenzyl group, which is known to react with cysteine through an S_N_Ar nucleophilic aromatic substitution-based mechanism (Fig 3A) [34]. Accordingly, to identify the putative covalent target of M4, we synthesized M4.67, a derivative of M4 bearing an alkyne moiety for use in affinity tagging and enrichment studies (Fig 3B). We next performed chemical proteomics enrichment studies to identify the proteins covalently labeled by M4.67. HEK 293T cells were exposed to 1 µM M4.67 for 1 h and lysates were subjected to click chemistry reactions to affix biotin azide to labeled proteins. Streptavidin enrichment in denaturing conditions, coupled to shotgun AP-MS/MS proteomic analysis, identified STARD7 as statistically enriched in the immunoprecipitate (S1A Table, Fig 3C,). M4.67 labeling of STARD7 was also confirmed in A549 cells, as 20 µM M4.67 treatment for 1 h followed by biotin azide click reaction and streptavidin enrichment resulted in robust detection of STARD7 by immunoblotting (Fig 3D).

To validate target engagement, HEK293T cells overexpressing FLAG-tagged STARD7 were treated with M4.67 (100 nM-100 µM) for 1 h, FLAG-tagged STARD7 immunoprecipitated, and then subjected to a click chemistry reaction with rhodamine azide. Rhodamine positivity of the immunoprecipitated STARD7 was found to increase in a concentration-dependent manner, confirming the chemoproteomic profiling results (Fig 3E).

We next sought to characterize the target residue/s of STARD7 that is covalently labeled by M4.67. We could specifically detect a tryptic peptide fragment adduct containing C302 by MS/MS from HEK293T cells overexpressing STARD7 treated with 20 µM M4.67 for 1 h. (S1B Table, Fig 3F,). Mature processed STARD7 contains two cysteines, C302 and C362 [35]. To probe the involvement of these cysteines in the interaction with M4, we generated cysteine mutants of FLAG-tagged STARD7 (C302S and C362S) and overexpressed them in HEK293T cells. After treatment with M4.67, FLAG immunoprecipitation, and click-reaction based conjugation to rhodamine azide, it was found that the C302S mutant no longer bound to M4.67, suggesting that this residue is likely the target of M4.67 (Fig 3G). Cysteine 302 of STARD7 is located within the START domain, which is essential for lipid binding, transport and metabolism [35]. It likely plays a critical role in the lipid-binding function of the protein, either by directly interacting with lipids or by maintaining the structural integrity of the lipid-binding domain.

### STARD7 is required for influenza A virus replication

**Fig 4:**
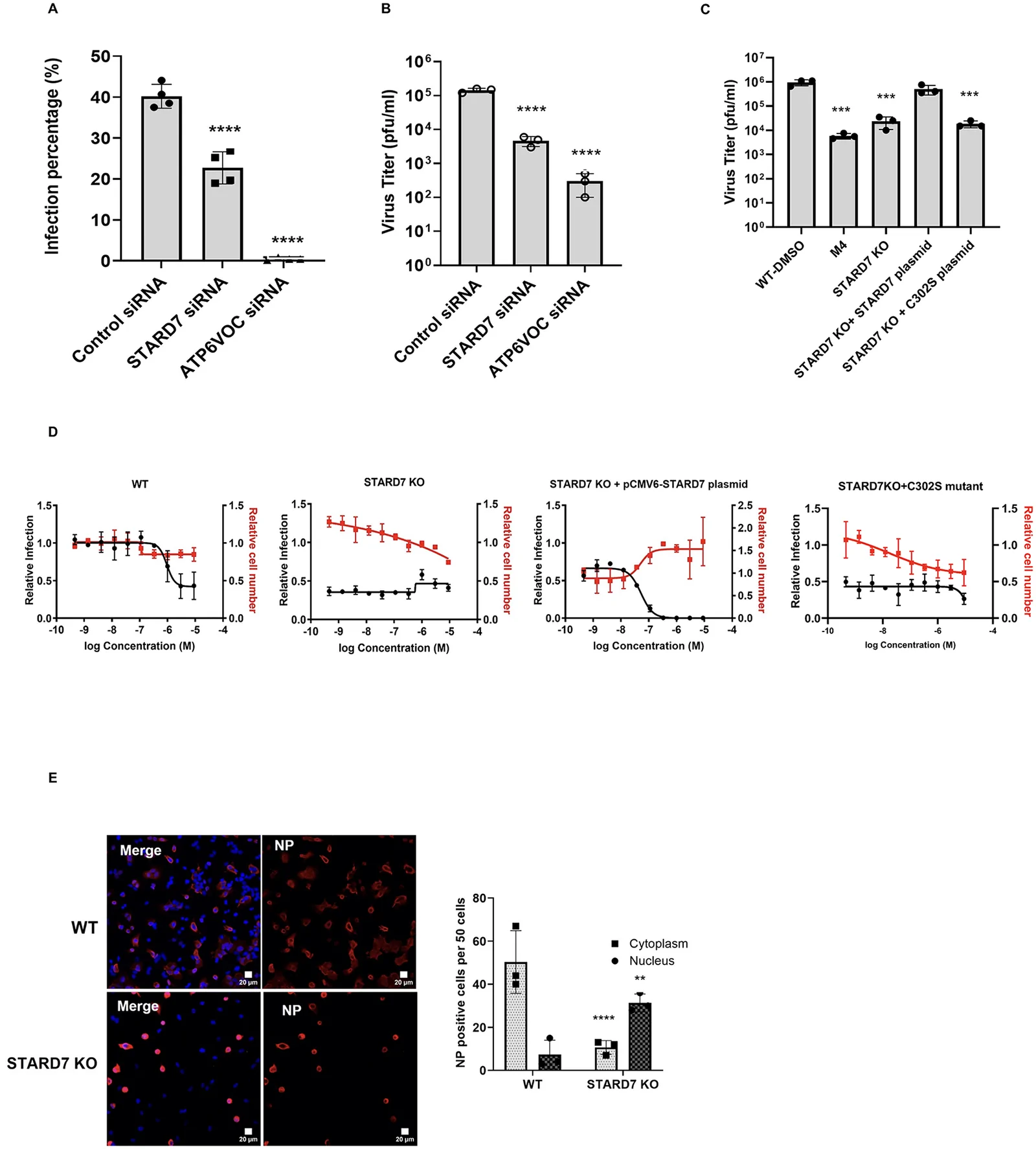
STARD7 is required for IAV replication, and M4 antiviral activity (A) A549 cells were transfected with non-targeting control siRNA or siRNA targeting STARD7 or ATP6V0C followed by infection with WSN at MOI of 0.01. At 32 h post infection, the cells were fixed, stained for NP and analyzed with a high content immunofluorescence imager. Percent infection is shown. Statistical significance was determined with a one-way ANOVA. ****p < 0.0001. **(B)** A549 cells were transfected with non-targeting control siRNA or siRNA targeting STARD7 or ATP6V0C followed by infection with WSN at MOI of 0.01. Titers were quantified by plaque assay at 24 post infection. Statistical significance was determined with a one-way ANOVA. **** p < 0.0001. **(C)** DMSO-treated WT A549 cells, 3 μM M4 treated WT cells, STARD7 KO cells, STARD7 KO  +  80 ng of transfected pCMV6-STARD7 and STARD7 KO  +  80 ng of transfected C302S plasmids cells were infected with WSN (MOI 0.25) for 48 h followed by quantification of titers by plaque assay. **(D)** WT A549 cells, STARD7 KO cells, STARD7 KO + transfected pCMV6-STARD7 plasmid and STARD7 KO + transfected pCMV6-STARD7 (C302S) cells were infected with WSN (MOI 0.25) and treated with M4. 48h post infection, cells were fixed, stained and analyzed with a high content immunofluorescence imager. Percent infection was calculated as the ratio of anti-NP-stained cells to DAPI stained cells. Data were normalized by the mean for DMSO-treated wells and represent means ± SEM from three independent experiments. Dose-response curves for infectivity (black) and cell number (red) are shown. **(E)** WT and STARD7 KO cells were infected with A/WSN/33 at an MOI of 3 for 16h. The cells were fixed, labelled with anti-NP and DAPI. Quantification of NP positive cells with nuclear and cytoplasmic staining where at least fifty cells per condition were quantified for each image with Image **J.**

To determine if STARD7 is a proviral host factor required for influenza A virus replication, gene-specific siRNA knockdown was optimized in A549 cells to achieve a > 90% reduction in STARD7 expression (S4A Fig). Infection of STARD7-depleted cells was evaluated by immunostaining of NP and a significant reduction (50%) was consistently observed (Fig 4A). To further validate this observation, virus titers were quantified by plaque assay in STARD7 knockdown cells. Virus release was significantly decreased (>1.5 log_10_) at 24 h post infection (Fig 4B). Next, we employed CRISPR-mediated genome editing to ablate STARD7 in A549 cells. WSN replication was diminished by approximately 1.5 log_10_ (p < 0.005) in these cells and was comparable to that in M4-treated A549 cells (Fig 4C). Importantly, complementation of knockout cells with wild-type STARD7 re-established viral replication to levels observed in parental A549 cells, while viral replication was not significantly recovered in knockout cells complemented with STARD7 C302S mutant (Fig 4C). Additionally, M4 did not exhibit antiviral activity in STARD7 knockout cells, nor those complemented with mutant C302S compared to wild-type STARD7 and interestingly, supplementing the knockout cells with the full-length STARD7 plasmid significantly restored antiviral activity of M4 (Fig 4D), further indicating that M4 likely exerts its antiviral activity though inhibition of STARD7. Finally, we find that STARD7 knockout, or knockdown, led to retention of vRNP in the nucleus, phenocopying antiviral activities seen with M4 treatment (Fig 4E, S5A Fig). These data strongly support STARD7 as the critical host protein that is targeted by M4 to exert its antiviral activity. Furthermore, it underscores the significance of STARD7 as a host factor that is essential for the correct nuclear export of vRNPs, and it provides functional evidence that proviral activities of STARD7 can be pharmacologically targeted to inhibit viral replication.

### *In vivo* evaluation of M4 in combination with direct-acting antivirals

**Fig 5:**
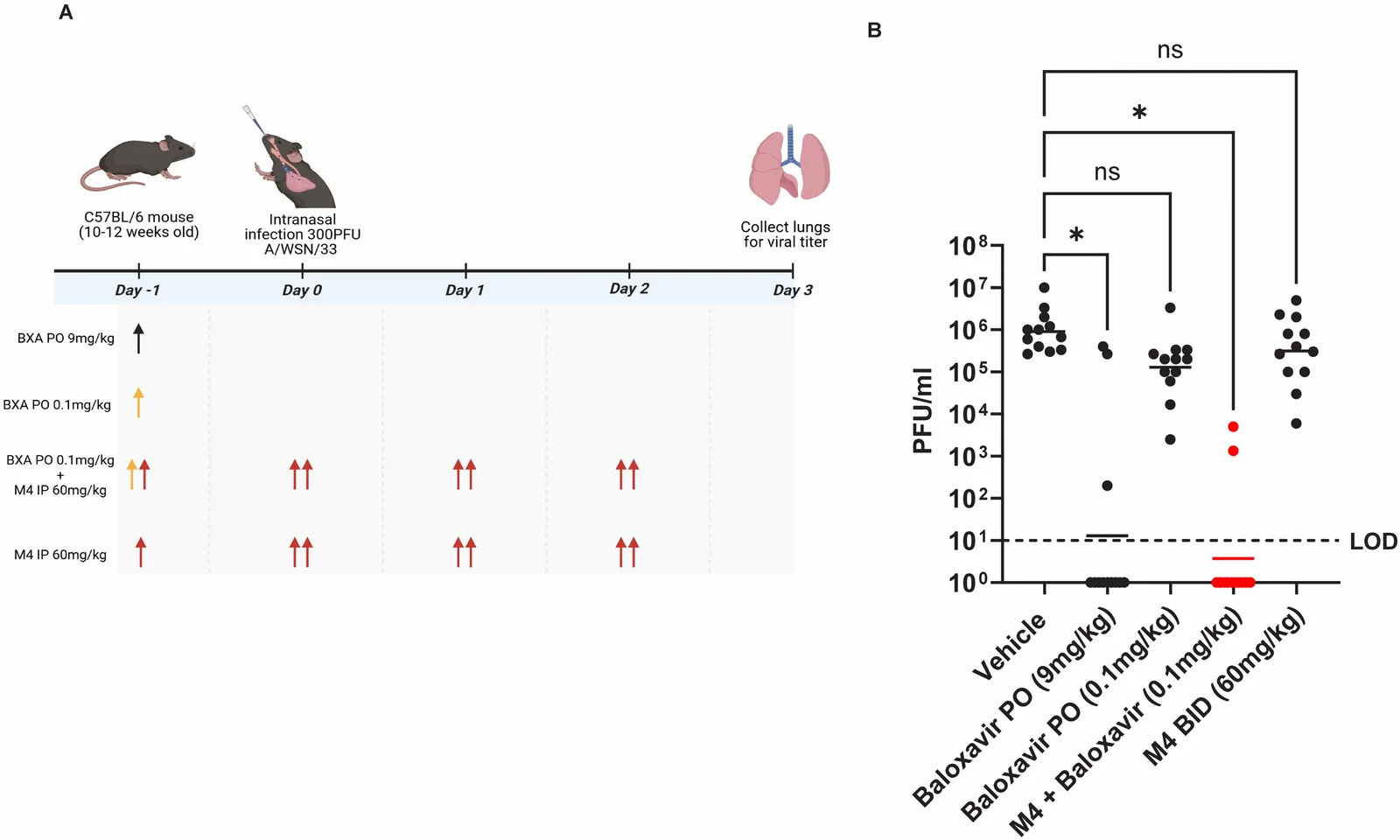
M4 demonstrates in vivo antiviral activity and enhances the efficacy of baloxavir in a mouse model of influenza A virus infection. **(A)** Schematic of the experimental design. C57BL/6 mice (10–12 weeks old) were infected intranasally with 300 PFU of A/WSN/33 (H1N1) on Day 0. Treatments were administered according to the indicated schedule: baloxavir (PO) at 0.1 or 9 mg/kg once daily, M4 (IP) at 60 mg/kg twice daily (BID), or combination therapy with M4 (60 mg/kg IP BID) plus baloxavir (0.1 mg/kg PO). Lungs were harvested on Day 3 post-infection for viral titer quantification. The schematic was generated using BioRender. **(B)** Viral loads were determined by plaque assay and are presented as PFU/mL on a log_10_ scale. The limit of detection (LOD) is indicated by the dashed line. Statistical significance was assessed by one-way ANOVA with appropriate post-hoc tests; ns = not significant.

To evaluate the antiviral efficacy of M4 in vivo, we performed a prophylactic treatment study in a murine model of influenza A virus infection. Mice were administered M4 intraperitoneally at 60 mg/kg twice daily, beginning one day prior to infection with influenza A/WSN/33 virus and continuing through day 3 post-infection (Fig 5A). This dosing regimen was informed by a prior maximum tolerated dose study, in which 60 mg/kg administered twice daily represented the highest dose tested that did not cause significant weight loss (S6A-C Fig). As monotherapy, even at near maximally tolerated doses, M4 had minimal impact on viral replication and body weight rescue, likely due to insufficient exposure, with lung titers of approximately 10⁶ PFU/mL comparable to those observed in vehicle-treated animals (Fig 5B and S6D).

Given the limited efficacy of M4 alone, we next evaluated its use in combination with a subtherapeutic dose of a direct-acting antiviral to enhance antiviral activity while limiting drug exposure and reducing the risk of resistance. Baloxavir marboxil (BXA) was selected for combination studies. BXA was administered prophylactically at 0.1 mg/kg, well below the calculated therapeutic dose of 9 mg/kg and alone did not significantly affect lung viral titers. In contrast, combination treatment with M4 and baloxavir reduced viral titers to below the limit of detection, consistent with an additive or potentially synergistic interaction and supporting further development of this host-directed combination strategy (Fig 5B).

## Discussion

In this study, we identify the small molecule M4 as a covalent inhibitor of influenza virus replication that targets the host lipid transfer protein STARD7. M4 exhibits antiviral activity against a broad range of influenza A viruses, including seasonal H1N1 and H3N2 strains and an avian H5N1 isolate, as well as influenza B virus, demonstrating broad activity across influenza viruses. Mechanistic studies reveal that M4 suppresses influenza virus replication by preventing formation of export-competent viral ribonucleoprotein (vRNP) complexes, resulting in nuclear retention of viral genomes and inhibition of progeny virus production. Time-course analyses further demonstrate only a modest and transient reduction in viral protein accumulation and vRNA levels following M4 treatment, whereas viral mRNA levels remain largely unaffected. Importantly, viral protein and vRNA levels recover at later stages of infection while the defect in vRNP nuclear export persists, indicating that impaired nuclear export is unlikely to be a secondary consequence of altered viral gene expression. At the same time, the transient effects on viral protein accumulation and vRNA raise the possibility that STARD7 contributes to additional stages of the viral replication cycle. Alternatively, these early changes may arise as a consequence of impaired vRNP assembly and nuclear export. Distinguishing between these possibilities will require further investigation. Using complementary chemoproteomic, genetic, and biochemical approaches, we identify STARD7 as the primary functional target of M4 and demonstrate that genetic depletion of STARD7 phenocopies the effects of pharmacologic inhibition on vRNP nuclear export. Although contributions from additional cellular targets cannot be completely excluded, the collective evidence supports STARD7 as the principal mediator of M4 antiviral activity. Together, these findings identify STARD7 as a previously unrecognized host factor required for efficient influenza virus replication and reveal a host-controlled metabolic checkpoint governing influenza vRNP assembly and nuclear export.

STARD7 is a member of the steroidogenic acute regulatory related lipid transfer domain protein family, whose members play critical roles in intracellular lipid transport, particularly involving cholesterol, phospholipids, and other sterols that are essential for membrane composition and signaling [36]. STARD7 is specifically involved in the transfer of phosphatidylcholine to mitochondrial membranes and is required to maintain mitochondrial membrane integrity and cellular respiration [37–39]. STARD7 exists in two isoforms: the precursor form STARD7-I, which contains a mitochondrial targeting sequence, and the shorter form STARD7-II, which results from cleavage of STARD7-I by mitochondrial proteases and is localized predominantly in the cytoplasm, plasma membrane, and nucleus [40,41]. STARD7-I is responsible for delivery of phosphatidylcholine to mitochondria, while STARD7-II has been implicated in cell migration through ERK1/2 signaling, connexin 43, and integrin β1 pathways [42]. The extra-mitochondrial form of STARD7 is also required for transport of coenzyme Q from mitochondria to the plasma membrane [43]. Because phospholipid transfer frequently occurs at mitochondria–ER membrane contact sites, which serve as hubs for lipid exchange and metabolic signaling, STARD7 is well positioned to couple lipid flux and mitochondrial state to downstream cellular processes. Although our chemoproteomic, genetic, and biochemical data identify STARD7 as the primary functional target of M4, the mechanisms by which STARD7 regulates influenza virus replication remain to be fully defined. In particular, future studies will be needed to determine the relative contributions of the mitochondrial and extra-mitochondrial STARD7 isoforms and to establish whether STARD7 regulates vRNP assembly and nuclear export through direct interactions with the viral replication machinery or indirectly through its phosphatidylcholine transfer activity.

We find several lines of evidence suggesting that M4 exerts its antiviral activity through inhibition of the lipid-transfer function of STARD7. Binding studies indicate that M4 binds within the lipid transferase pocket of STARD7. Mutation of cysteine 302 to serine in this domain abolished M4 binding, and the C302S mutant failed to rescue influenza replication in STARD7-deficient cells, whereas wild-type STARD7 restored replication. These data place the STARD7 lipid-transfer domain, rather than a scaffolding or signaling role alone, at the center of its proviral function. Importantly, M4 treatment phenocopies genetic depletion of STARD7, including nuclear retention of vRNPs, supporting the conclusion that M4 defines a functional checkpoint controlled by STARD7 lipid handling.

Influenza vRNP nuclear export requires assembly of a complex containing NP, M1, and NEP, which subsequently engages the CRM1 export machinery [29,30]. While CRM1 has been well characterized in this process, the host determinants that contribute to formation of export-competent vRNP complexes remain incompletely understood. Prior work has shown that influenza hemagglutinin–mediated signaling activates the Raf–MEK–ERK–RSK pathway, which promotes phosphorylation of NP and facilitates its interaction with M1, thereby licensing vRNP nuclear export [31]. Critically, we find that M4 inhibits vRNP nuclear export without phenocopying CRM1 inhibition and instead disrupts the association of NP with M1 and NEP, suggesting that STARD7 functions upstream of CRM1 engagement, and to promote formation of an export-competent vRNP complex. In addition to NP, M4 treatment promoted nuclear accumulation of M1. Because M1 enters the nucleus and associates with vRNPs and NEP prior to CRM1-dependent export, this phenotype is consistent with impaired assembly and/or export of the vRNP complex. Whether STARD7 regulates these events through direct interactions with viral components or indirectly through its established role in phosphatidylcholine transport and cellular lipid homeostasis remains to be determined. Likewise, defining the relative contributions of the mitochondrial (STARD7-I) and extra-mitochondrial (STARD7-II) isoforms to vRNP assembly and nuclear export will be an important direction for future investigation. Together, our findings identify STARD7 as a previously unrecognized host factor linking cellular lipid homeostasis to influenza vRNP assembly and nuclear export.

Our findings reveal an unexpected role for STARD7 in regulating influenza vRNP nuclear export thereby expanding the known biological functions of this mitochondrial lipid transfer protein. These observations raise intriguing mechanistic questions regarding how STARD7 is functionally coupled to the viral nuclear export machinery, including whether this occurs through direct interaction with viral or host factors or indirectly through its phosphatidylcholine transfer activity and the downstream cellular pathways it regulates. Elucidating these mechanisms, together with the spatiotemporal dynamics and isoform-specific functions of STARD7 during infection, will further define its role in the influenza virus life cycle. One possibility is that STARD7 regulates vRNP export indirectly through mitochondrial signaling pathways. Inhibition of the Raf–MEK–ERK cascade also results in nuclear retention of vRNPs, a phenotype mediated by the redox-sensitive kinase RSK1, which phosphorylates NP and promotes its interaction with M1 [44–46]. Because STARD7 contributes to mitochondrial lipid handling and cellular redox balance, changes in its activity could modestly influence signaling pathways such as RSK1 that participate in NP phosphorylation. These models are not mutually exclusive and point to an integrated role for lipid metabolism, redox signaling, and viral trafficking in controlling influenza nuclear export. Alternatively, host lipid metabolism is upregulated during influenza infection, and the viral matrix protein M1 directly interacts with phosphatidylcholine, phosphatidylserine, and cholesterol and has been implicated in vRNP nuclear export [47,48]. One possibility is that STARD7-dependent phosphatidylcholine trafficking supports the lipid environment required for stable M1–NP–NEP complex assembly, and that inhibition, or loss, of STARD7 perturbs this lipid-dependent step.

Targeting host pathways required for viral replication offers the advantage of a higher barrier to resistance compared with direct-acting antivirals. Consistent with this, we did not observe resistance to M4 after extended viral passaging. Importantly, M4 does not disrupt CRM1-dependent export of host proteins, suggesting a selective mechanism that may limit toxicity relative to direct CRM1 inhibitors. Although M4 alone showed limited efficacy *in vivo* at near-maximally tolerated doses, combination with a subtherapeutic dose of baloxavir resulted in complete suppression of viral replication in a murine model. This provides *in vivo* proof of concept that partial inhibition of a host metabolic checkpoint can strongly potentiate direct-acting antivirals. Such combination strategies may enable dose sparing, improved efficacy, and reduced emergence of resistance.

In summary, this work identifies STARD7 as a metabolic and signaling node that links phospholipid transfer to influenza vRNP assembly and nuclear egress. By demonstrating that covalent engagement of the STARD7 lipid-transfer domain blocks vRNP export and viral replication, these findings establish lipid regulation as a previously unrecognized checkpoint in the influenza life cycle and highlight STARD7 as a tractable target for host-directed antiviral discovery.

## Materials and methods

### Ethics statement

All experiments were conducted under an Institutional Animal Care and Use Committee (IACUC)-approved protocol (24-0006-1; 07/01/2022-06/30/2032; IACUC at The Scripps Research Institute) in a USDA-registered research facility.

### Cells and viruses

A549 (ATCC CCL-185), MDCK (ATCC CCL-34), and MEF (ATCC CRL-2991) cells were cultured at 37°C in a humidified incubator with 5% CO2 in DMEM (Gibco, Life Technologies) supplemented with 10% FBS (Gibco, Life Technologies), 1 mM sodium pyruvate (Gibco, Life Technologies), 10 mM HEPES (Gibco, Life Technologies), and 100 U/ml of penicillin and 100 μg/ml of streptomycin (Gibco, Life Technologies). HTBE cells (ATCC PCS-300–010) were cultured in commercially available airway epithelial cell basal media following the manufacturer’s protocol (ATCC). HeLa S3 (ATCC CCL-2.2) cells were cultured at 37C in a humidified incubator with 5% CO2 in DMEM/F-12 (Gibco, Life Technologies) supplemented with 10% FBS and 100 U/ml of penicillin and 100 μg/ml of streptomycin (Gibco, Life Technologies). A549-doxycycline(dox)-Cas9 cells were generated by transduction with Lenti-dox-Cas9 (Dharmacon) into A549 cells. Following transduction, cells were plated for colony formation and screened for Cas9 expression after doxycycline treatment. Cell viability was assessed using DAPI staining with a high content imager.

Influenza A/WSN/33 (H1N1), A/Wyoming/3/03 (H3N2), A/California/07/2009, A/Vietnam/1203/04-HALo and B/Yamagata/88 viruses were propagated in MDCK cells. A/Vietnam/1203/04 (H5N1) HALo mutant virus is an attenuated H5N1 influenza A virus that lacks the polybasic cleavage site in HA [49]. DENV-2 (Nicaragua isolate) was propagated in C6/36 cell line. Poliovirus serotype 1, CHAT (ATCC VR-1562) was propagated in HeLa S3 cells.

### siRNA knockdown

Adenocarcinoma human alveolar basal epithelial cells, A549, were grown at 37°C in a humidified 5% CO_2_ atmosphere in DMEM supplemented with 10% FBS (Gibco, Thermofisher), penicillin, streptomycin, and amphotericin B mix (PAN-Biotech). A549 cells were transiently transfected with a pool of siRNA (40 nM siRNA/24-well) using Lipofectamine RNAiMAX (Invitrogen, Thermofisher) according to manufacturer's instructions. All RNA oligonucleotides were synthesized by Dharmacon Reagents (Lafayette, CO, USA). 48 h post-transfection, cells were infected with influenza A/WSN/33 (WSN) at MOI 0.01 (Fig 4A, B) or 6 (Fig 4E, S5A Fig) for 1 h at 4°C.

siRNA sequences used were:

**Table:** 

siNP	5’- GGAUCUUAUUUCUUCGGAGTT - 3’ 5’- CUCCGAAGAAAUAAGAUCCTT - 3’
siNT	DharmaconOn-target plus siControl nontargeting siRNA
siSTARD7	5’- CAUCGAUUGUGCAGCGCUU – 3’ 5’ – CCUCUGAGCGAAAGAACGA – 3’ 5’ - UUAAUGAGAUGAAGCGGUU - 3’ 5’ – CCAAUGUACUCACGGGAUU – 3’
siATP6VOC	5’-GGCACAGCCAAGAGCGGUA-3’ 5’-GCUCUGUGUAUGCGGAUGA-3’ 5’-CCCGACUAUUCGUGGGCAU-3’ 5’-CCAGCUAUCUAUAACCUUA-3’ 5’-GGGAGAAGAUGAUGACUAA-3’

### Antibodies for immunofluorescence

Rabbit anti-STARD7 polyclonal antibodies (ab221569, Abcam), mouse monoclonal anti-NP HT103 (Mount Sinai, in-house antibody), influenza A M1 monoclonal antibody (MA1–80736, Thermo Fisher Scientific), rabbit anti-PA (GTX11899, GeneTex), rabbit anti-PB1 (GTX125923, GeneTex), rabbit anti-PB2 (GTX125926, GeneTex), RanBP1 (ab97659, Abcam), anti-Flavivirus Group Antigen Antibody, clone D1-4G2-4–15 (MAB10216-I-25UG, Sigma) and Alexa Fluor 488-conjugated and 568-conjugated goat anti-mouse, Alexa Fluor 488-conjugated goat anti-rabbit secondary antibodies (A-11001 and A-11004, A-11008, Thermo Fisher Scientific).

### Antibodies for immunoblotting

Mouse anti-GAPDH (GTX627408, GeneTex), rabbit influenza nucleoprotein (GTX125989, GeneTex), rabbit influenza matrix 1 (GTX125928, GeneTex), rabbit influenza NS2/NEP (GTX125952, GeneTex), PARP1 (F-2) (sc-8007, Sant Cruz), rabbit influenza PA (GTX118991, GeneTex), rabbit influenza PB1 (GTX125923, GeneTex), rabbit influenza PB2 (GTX125926, GeneTex), Influenza A NS1 Polyclonal Antibody (PA532243, Invitrogen), HA (PA5–34929, Invitrogen), Biotin (ab53494, Abcam), STARD7 (PA5–112822, Thermo Fisher), FLAG (F3165, Sigma), Goat Anti-Rabbit IgG (H + L)-HRP Conjugate (1706515, BIO-RAD), Goat Anti-Mouse IgG (H + L)-HRP Conjugate (1706516, BIO-RAD), IRDye 680RD Donkey anti-Mouse IgG Secondary Antibody (926–68072, LICORbio), IRDye 800CW Donkey anti-Rabbit IgG Secondary Antibody (926–32213, LICORbio).

### High content imaging

A549, MEF, and HTBE cells were seeded in 384-well plates at a density of 5000 cells/well and incubated overnight in serum-free media pre-spotted with compounds. The following day, cells were infected with influenza virus at the indicated MOIs and at 48 h post-infection, cells were fixed with 10% PFA for 30 min and permeabilized with 0.5% Triton X-100 for 10 min. Blocking was performed with 3% BSA for 1 h, followed by incubation for 2 h at room temperature with primary anti -NP antibody at 1:3000 dilution. After three phosphate-buffered saline (PBS) washes, the cells were incubated with an Alexa Fluor 488-conjugated anti-mouse secondary antibody at a 1:1000 dilution. Following three additional PBS washes, plates were stained with DAPI and imaged using a high content imaging system IXMC. Images were acquired and analyzed using MetaXpress software for multi-wavelength cell scoring analysis. All experiments were performed in triplicate, and DAPI and FITC fluorescence signals were quantified for each condition. Relative cell number was expressed as the number of DAPI-stained nuclei in each well normalized to the average nuclei count of at least 20 DMSO (0.01%) control wells on the same 384-well plate.

### Subcellular trafficking and localization studies

**NP, PA, PB1, PB2, PA, & RanBP1 localization (S2A Fig):** A549 cells were seeded in glass-bottom chambered slides (Nunc) and incubated overnight in 10% FBS DMEM media at 37 °C and 5% CO2. Cells were washed once with 1 × PBS and infected with A/WSN/33 at an MOI of 3 indicated in S2A Fig for 1h. Inoculum was removed and replaced with M4 3 μM in serum-free media. At indicated time points, cells were washed twice with 1 × PBS and fixed using 4% paraformaldehyde (Fisher Scientific) for 30 min at room temperature. Cells were permeabilized using 0.5% Triton X-100 for 15 min, followed by 1 h of blocking with 3% BSA at room temperature. This was followed by immunolabelling with indicated primary and secondary antibodies. The slides were mounted on coverslips with Prolong anti-fade DAPI (Life Technologies) and sealed. Images were acquired using the 60x magnification of Zeiss LSM780 confocal microscope at The Scripps Research Institute Microscopy facility and localization was assessed using Fiji software.

**NP and M1 protein localization (Fig 2B, and S2B, S5A Fig):** MDCK or A549 cells were seeded on glass coverslips in a 24-well plate and grown to 80% confluence overnight at 37°C and 5% CO_2_ in DMEM supplemented with 10% FBS (Gibco, Thermofisher), 1% penicillin, streptomycin, and amphotericin B mix (PAN-Biotech). Cells were pre-treated with post-inoculation media (DMEM, 0.3% BSA, 0.1% FBS, 1% penicillin, streptomycin, and amphotericin B mix) containing either M4 (10 µM for NP localization and 3 µM for M1 localization) or DMSO for 2 h at 37°C. Cells were washed with 1X PBS and infected with A/WSN/33 (MOI of 5 for NP localization and MOI of 3 for M1 localization) for 1 h at 4°C in the presence or absence of M4. Virus inoculum was aspirated, cells were washed with 1X PBS, and replenished with post-inoculation media containing M4 and TPCK-treated trypsin (1 µg/mL); thereafter cells were incubated for a total of 4, 6, 8, and 12 hpi. At the indicated times post infection, cells were fixed in 100% methanol for 30min at -20°C. Cells were washed twice with 1X PBS followed by a 2 h block with 5% BSA at RT. Subsequently, cells were immunostained with anti-NP mouse monoclonal (HT103) or anti-M1 mouse monoclonal (MA180736) (1:2000) for 1 h at RT. Hereafter, cells were washed with 1X PBS-Tween 20 and incubated with Alexa Fluor 488-conjugated anti-mouse secondary antibody (1:500) and DAPI (1:1000). The coverslips were mounted on slides with ProLong Glass antifade (P36982). Images were acquired using the Nikon Eclipse 50i fluorescence microscope running NIS-Elements software (version BR 4.20.03) and further processed using ImageJ (version 1.53t) software.

**STAT1-GFP trafficking** **(S2H Fig):** Vero E6 cells in a 24-well plated were transiently transfected with 200 ng STAT1-GFP plasmid (provided by Prof. Megan Shaw (University of the Western Cape, Cape Town, South Africa and described elsewhere [50] DNA using Lipofectamine 3000 (Invitrogen, Thermofisher) according to manufacturer's instructions. 24 h post-transfection, cells were stimulated with interferon-β (IFNβ; 1000 U/mL; IF014, Merck) in complete media supplemented with cycloheximide (CHX; 10 µg/mL; C7698, Merck) and containing either DMSO, M4 (10 μM), or leptomycin B (50 nM). The subcellular localization of STAT1-GFP was determined by live-cell imaging at 10 min, 1 h, and 5 h post-stimulation. Images were acquired using the Zeiss Primovert microscope equipped with the Axiocam 208 colour camera.

### Time-of-addition assay

MDCK cells were plated in complete DMEM and grown to 80% confluence for 24 h. Cells were then treated with 3 µM M4 at 0, 2, 4, 6, 8 h pre/post infection with influenza A/WSN/33 virus (MOI 0.1). Supernatants were collected 24 h post infection and viral titers were quantified by plaque assay.

### Influenza virus entry assay

Replication-incompetent, vesicular stomatitis virus (VSV) engineered to express green fluorescent protein (GFP) and firefly luciferase (FLuc) in lieu of the viral glycoprotein (G), was kindly gifted by Dr. Gert Zimmer at the Institute for Virology, Mittelhäusern, Switzerland. VSV pseudoviruses bearing the HA and NA protein of influenza A/WSN/33 virus were generated by transfecting HEK-293T cells with plasmids expressing HA and NA, and then infecting the cells with VSVΔG(FLuc) that had been complemented with VSV G. Supernatants containing VSVppHA/NA were collected and titrated based on firefly luciferase expression.

For the influenza entry assay, MDCK cells were pre-treated with DMSO, M4, or S20 for 2 h before infection with VSVppHA/NA in the presence of compounds [28]. Luciferase activity in the cell lysates was measured at 24 h post infection and expressed relative to the DMSO control.

### Resistance study

A549 cells were infected with WSN at an MOI of 0.01 and maintained in the presence of DMSO, M4 treatment (at IC90; 1.06 μM) or BXA (at IC90; 6 nM) for 48 h at 37 °C. The culture supernatant was then harvested, and titered by plaque assay. The resulting virus was used to infect fresh A549 cells under the same treatment conditions for a total of 10 serial passages. Following passage 10, antiviral susceptibility was assessed by plaque reduction assay using M4 (at 1.2, 2.5, 5, and 10 μM for DMSO- and M4-passaged virus) or BXA (at 1.2, 2.5, 5, and 10 nM for DMSO- and BXA-passaged virus), and IC50 values were determined by nonlinear regression analysis in GraphPad Prism.

### Nuclear and cytoplasmic fractionation

A549 cells seeded in 6-well plate and next day, pre-treated with 1.06 μM M4 for 16 h, followed by infection with WSN at 1 MOI for 12 h. Nuclear and cytoplasmic fractions were isolated using the NE-PER Nuclear and Cytoplasmic Extraction Reagents (Thermo Fisher Scientific) according to the manufacturer's instructions. Each fraction was divided equally for immunoblotting and RT-qPCR analyses. Fraction purity was validated by immunoblotting using compartment-specific marker proteins. Bands were visualized using LI-COR instrument, image studio software.

### RNA extraction and RT-qPCR

For strand-specific detection of viral RNA in cytoplasmic and nuclear fractions, primers were designed for influenza virus A positive-sense mRNA and negative-sense vRNA, each containing an additional unrelated 18- to 20-nucleotide tag at the 5′ end to enhance specificity and differentiate between the RNA species, as described previously [51]. Briefly, equal amounts of fractionated RNA were reverse-transcribed into cDNA using the Applied Biosystems cDNA synthesis kit. qPCR was then performed using the RT2 SYBR Green qPCR Master Mix (Applied Biosystems) with primer sets specific for the corresponding influenza virus RNA [51] on an ABI thermocycler. Viral RNA abundance was normalized to GAPDH for cytoplasmic fractions and U6 for nuclear fractions (Fig 2C) or 18S rRNA for quantifying the NP, NA and M vRNA and mRNA levels (S2E Fig). Relative expression levels compared to mock-treated samples were calculated using the 2^−ΔΔCT^ formula [52].

### Generation of CRISPR-Cas9 STARD7 knockout (KO) cells

To generate STARD7 knockout (KO) cells using CRISPR-Cas9, we targeted the STARD7 locus with the sequence ACCCTCCAGAACCAAAAGCC and generated a full-length guide RNA (gRNA) using a gRNA synthesis kit (Thermo Fisher). The gRNA was transfected into A549-dox-Cas9 cells that had been pre-treated with doxycycline (1 μg ml–1; Clontech) for 48 h to induce Cas9 expression. At 48 h post-transfection, cells were plated for single-cell colony formation. Colonies were screened via western blot analysis to confirm STARD7 knockout. Cell viability was assessed by Western blot for STARD7 (PA5–30772, Thermo Fisher) and β-actin (4967, Cell Signaling), and cell number quantified with DAPI staining with a high content imager.

### Streptavidin enrichment studies

For streptavidin enrichment studies, confluent A549 cells grown in a 10-cm tissue culture dish were washed twice with phenol red-free DMEM (Gibco). The cells were then treated with 20 µM M4.67 or an equivalent volume of DMSO in phenol red-free DMEM without FBS and incubated at 37 °C for 1 h. Following treatment, cells were washed twice with PBS, scraped into 1 mL PBS, and then lysed by sonication. Insoluble material was removed by centrifugation. Two 1.5-mL microcentrifuge tubes, each containing 0.5 mL of lysate (2 mg/mL), were incubated with a Click reagent mix consisting of 30 µL of 1.7 mM TBTA in tBuOH:DMSO 4:1, 10 µL of 50 mM CuSO_4_ in H_2_O, 10 µL of 50 mM TCEP in H2O, 2.5 µL of 20 mM biotin-PEG3-N_3_ at room temperature for 1 h. Samples were then precipitated in ice-cold methanol, and the resulting pellet was resuspended in 1 mL PBS containing 0.6% sodium dodecyl sulfate (SDS). The resuspended lysates were pooled into a 2-mL mixture and incubated for 24 h at 4 °C with 220 µL streptavidin beads in 10 mL PBS on a rotator. Beads were pelleted by centrifugation (2,000 x*g* for 1 min) and subjected to sequential washes: two washes with 10 mL of 0.1% SDS in PBS, two washes with 10 mL of PBS twice, and two final washes with 10 mL of water. After washing, 200 µL of SDS-PAGE sample buffer supplemented with 10% beta-mercaptoethanol was added to the beads, and the beads were boiled at 99 °C for 15 min. The resulting supernatant was analyzed via western blot using anti-biotin and anti-STARD7.

### Immunoprecipitation studies

Plasmids encoding FLAG-tagged STARD7 were obtained from Origene (RC202539). Cysteine mutants were generated using site-directed mutagenesis (NEB E0554S). For immunoprecipitation studies using FLAG-tagged transgenes, HEK293T cells (5  ×  10^6^) were transfected with 2 µg of each plasmid per well of a six-well plate using 100 µL of OptiMEM medium (Gibco) containing 8 µL of FuGENE HD transfection reagent (Promega). After 24 h, the growth medium replaced with fresh medium, and cells were incubated for another 24 h. At 48 h post-transfection, cells were treated with 20 µM M4.67 in serum-free DMEM for 1 h. Following treatment, cells were washed twice with PBS, scraped into 250 µL of ice-cold RIPA buffer (EMD Millipore), and lysed by sonication. Insoluble material was removed by centrifugation, and protein concentration was determined via absorbance measurements. For immunoprecipitation, 1mg of total lysate in (1 mL of RIPA buffer) was incubated overnight at 4 °C with 20 µL of anti-FLAG M2 magnetic bead slurry (Sigma). Beads were washed three times with 300 µl of RIPA, and bound protein complex were eluted using 250 µg/mL of FLAG peptide (DYKDDDDK, Sino Biological) in PBS. Eluted samples were then subject to click reaction (described previously) and incubated at room temperature for 1 h. The reaction mixture was precipitated in ice-cold methanol, and the pellet was resuspended in 50 µL SDS-PAGE sample buffer with 10% beta-mercaptoethanol. Immunoprecipitated proteins were separated by SDS-PAGE and analyzed using a ChemiDoc MP imager (Bio-Rad) via rhodamine fluorescence scanning and anti-FLAG immunoblotting.

To examine NP interactions with viral proteins during infection, A549 cells were seeded in 10-cm dishes, pre-treated with M4 (5 µM) or DMSO for 16 h at 37 °C, and infected with influenza A/WSN/33 at an MOI of 3 for 1 h at 4 °C. Following virus adsorption, inoculum was removed, and cells were incubated in infection medium containing M4 (5 µM) or DMSO for 8 h at 37 °C.

Cells were washed with ice-cold PBS and lysed in ice-cold Pierce IP lysis buffer supplemented with protease and phosphatase inhibitors. Lysates were clarified by centrifugation at 5,000 × g for 20 min at 4 °C. A fraction of each lysate was retained as input. Remaining lysates were incubated overnight at 4 °C with Protein G magnetic beads pre-bound to anti-influenza A nucleoprotein antibody (GTX125989, GeneTex).

Beads were washed three times with Pierce IP lysis buffer, and bound proteins were eluted in 1 × Laemmli buffer by boiling at 95 °C. Input and immunoprecipitated samples were resolved by SDS-PAGE and transferred to PVDF membranes (Immobilon-P, Merck). Membranes were blocked in 3% BSA in TBST and probed with antibodies against influenza A NP (GTX125989, GeneTex,), M1 (GTX125928, GeneTex), NS2/NEP (GTX125952, GeneTex,), and GAPDH (GTX627408, GeneTex,), followed by HRP-conjugated secondary antibodies. Proteins were detected using enhanced chemiluminescence (ECL Prime, Cytiva).

### Animal studies

For the *in vivo* studies, 11–12-week-old male or female C57BL/6 mice (Jackson Laboratories, Bar Harbor, ME, USA) were randomly assigned to experimental groups. All procedures and handling adhered to the current standards specified in the Guide for the Care and Use of Laboratory Animals, and all experiments were conducted under an Institutional Animal Care and Use Committee (IACUC)-approved protocol (24-0006-1; 07/01/2022-06/30/2032; IACUC at The Scripps Research Institute) in a USDA-registered research facility. The mice were bred and housed in a BSL2 barrier facility following institutional guidelines. In these studies, mice were divided into experimental groups of 3 and received different treatments. Vehicle (0.2% methylcellulose and Tween 80) and baloxivir was administered orally once 12 h prior to infection, while M4 was given intraperitoneally twice daily, starting 12 h before infection and continuing until day 4 post-infection. For virus challenges, mice were anesthetized using the open-drop method of isoflurane exposure and then received an intranasal administration of 300 PFU of influenza A/WSN/33 (H1N1) in a 30 μL volume. On day 5 post-infection, 5 mice were euthanized, and their lungs were collected, homogenized in 1 mL of DMEM, and stored at −80 °C until titration was performed using the standard plaque assay in MDCK cells. The remaining 3 mice were monitored for body weight for 5 days.

## Supporting information

S1A Fig MDCK cells were infected with influenza HA/NA-pseudotyped VSV particles in the presence of DMSO, M4, or the HA fusion inhibitor S20. Luciferase activity was measured at 24 h post-infection to assess entry efficiency. The experiment was performed in triplicate and the means + /- standard deviation are shown.
(TIF)

S2AB Fig A549 cells were pre-treated for 2 h with 3 μM M4 followed by infection with A/WSN/33 at 3 MOI for 24 h. Cells were then fixed, labeled with anti NP, PA, PB1, PB2, or RanBP1 antibodies and analyzed for immunofluorescence. Representative images are shown. Quantification in the last panel of each row was done with Image J. S2B Fig. Subcellular localization of influenza A virus M1 protein at indicated times post-infection in M4 treated cells. MDCK cells treated with M4 (3 µM) and infected with influenza A/WSN/33 virus (MOI 3) were immunolabeled with anti-M1 antibodies at 4, 6, 8, and 12 hpi to capture the cellular trafficking of M1 proteins. Scale bar = 20 µm. Five fields of view were randomly selected for M4 and DMSO, respectively. The localization of M1 was recorded as present either in the “nucleus” or “nucleus/cytoplasm” and graphically displayed as a percentage (%).
(TIF)

S2CDE Fig A549 cells were pretreated for 16 h with DMSO or 1 µM M4 followed by mock infection (UI) or infection with influenza virus A/WSN/33 at an MOI of 1 for 12 h. The cells were then subjected to cytoplasmic-nuclear fractionation, followed by immunoblotting from each fraction. Cytoplasmic and nuclear fraction cell lysates were analyzed by SDS-PAGE and immunoblotting for viral NP protein. GAPDH and PARP1 served as cytoplasmic and nuclear fraction control, respectively. S2D and S2E Fig: A549 cells were pre-treated for 16 h with 1 μM M4 followed by infection with A/WSN/33 at 1 MOI. Cells were then lysed at 4, 8, 12 and 24 hpi and were analyzed by SDS-PAGE and immunoblotting for viral proteins (S2D) or subjected to RNA isolation, followed by qPCR using fragment specific primers (S2E). GAPDH served control (S2D) while 18S rRNA served as internal control for qPCR data normalization (S2E).
(TIF)

S2FG Fig M4 antiviral activity against DENV (F) and poliovirus (G). Huh7.5.1 cells were pre-treated with M4 (40 – 0.1 μM) for 12 hr, followed by infection with DENV-2 at 0.2 MOI for 72 h. Cells were fixed and stained with 4G2 antibody and cell viability was measured using CellTiter-glo (F). HeLaS3 cells were plated in assay-ready plates with M4 or DMSO in assay media (growth media supplemented with 2% FBS) 4 h prior to infection with poliovirus PV-1 CHAT at MOI of 0.03 for 48 h, followed by measuring cytopathic effect with CellTiter-glo. In parallel, a matched uninfected HeLa S3 cytotoxicity assay was performed (G). The infection was normalized to untreated DMSO control.
(TIF)

S2HI Fig Vero E6 cells transiently expressing STAT1-GFP are shown in the presence of DMSO, M4 (10 µM), or Leptomycin B (LMB). Cells were either unstimulated or stimulated with IFNβ, and live-cell images of the subcellular localization of STAT1-GFP were collected by fluorescent microscopy at the indicated time points (1 h, and 5 h). *Scale bar = 25 µm*. For quantification, 29 fields of view were randomly selected for M4 and LMB, respectively. The localization of STAT1-GFP was recorded as present either in the “nucleus” or “nucleus/cytoplasm”, and the data are displayed as a percentage (%). S2I Fig. vRNA and mRNA associated with NP immunoprecipitates were quantified by RT-qPCR. A549 cells were infected with influenza A virus (WSN) and treated with either DMSO or M4 (1 µM). NP–vRNP complexes were isolated by immunoprecipitation, and viral RNA levels were measured using segment-specific primers. Ct values were converted to relative quantities using the 2^–ΔCt method with uninfected cells serving as the background reference. The analysis was performed to compare the relative recovery of vRNA and viral mRNA associated with NP immunoprecipitates. Data represent fold relative vRNA (left) and viral mRNA (right) associated with NP immunoprecipitates and are presented as the mean ± SEM from three technical replicates.
(TIF)

S3ABC Fig Schematic of the serial passaging workflow. The schematic was generated using ChatGPT (OpenAI). S3B and S3C Fig: Following 10 passages, A549 cells were infected at 0.01 MOI with DMSO- or M4-passaged influenza A/WSN/33 viruses and treated with increasing concentrations of M4 (1.2, 2.5, 5, and 10 μM) (S3B), or infected with DMSO- or BXA-passaged viruses and treated with increasing concentrations of BXA (1.2, 2.5, 5, and 10 nM) (S3C) for 48 h. Viral titers were determined by plaque assay, and IC50 values were calculated by nonlinear regression analysis in GraphPad Prism.
(TIF)

S1AB Table Top enriched proteins as high-priority target s. S1B Table: b and y ion designations from the modified tryptic STARD7 peptide containing C302, as shown in Fig 3G.
(TIF)

S4A Fig STARD7 knockdown efficiency by qPCR. A549 cells were transfected with either non-targeting (NT) siRNA or STARD7 siRNA, and mRNA levels were quantified by qPCR using STARD7-specific primers. Data are normalized to a GAPDH housekeeping gene and presented as fold change relative to NT siRNA. Statistical significance was determined with a student t-test. ****p < 0.01.
(TIF)

S5A Fig Subcellular trafficking of influenza A virus NP in STARD7 knockdown cells. A549 cells were transfected with no siRNA, non-targeting control siRNA, STARD7-targeting siRNA, or NP-targeting siRNA for 48 h, followed by infection with influenza A/WSN/33 virus. Subcellular localization of NP was captured by indirect immunofluorescence microscopy at 6-, 8-, and 12-h post-infection. Scale bar = *20 µm.* An average of 70 cells were quantified per condition at each timepoint. NP localization was scored as present either “in the nucleus” or “both in the nucleus and cytoplasm (nucleus/cytoplasm)” and the ratios are graphically shown as a percentage (%).
(TIF)

S6ABC Fig Schematic of the dosing regimen for the M4 maximum tolerated dose study. Mice were administered M4 intraperitoneally (IP) twice daily (BID) for six consecutive days at doses of 30 mg/kg, 60 mg/kg, or 100 mg/kg. The vehicle was 50% PEG-400 in PBS. The schematic was created using BioRender. S6B Fig: Body weight was recorded daily and expressed as a percentage of baseline (Day 0). Mice receiving 100 mg/kg exhibited weight loss exceeding 20% of starting body weight by Day 3, triggering humane euthanasia per IACUC guidelines.S6C Fig: Kaplan–Meier survival analysis over the 6-day treatment period.
(TIF)

S6D Fig Body weight was recorded daily during the infection period. (TIF)
